# NCAPG Promotes Tumor Progression and Modulates Immune Cell Infiltration in Glioma

**DOI:** 10.3389/fonc.2022.770628

**Published:** 2022-03-15

**Authors:** Guangrong Zheng, Tao Han, Xiaomu Hu, Zhou Yang, Jin Wang, Zhenyi Wen, Hengyu Li, Hongjin Wang

**Affiliations:** ^1^ Department of Neurology, The Second Hospital of Dalian Medical University, Dalian, China; ^2^ Department of Radiology, Changhai Hospital, Navy Medical University, Shanghai, China; ^3^ Department of Oncology, The First Affiliated Hospital of China Medical University, Shenyang, China; ^4^ Department of Pathology, Huashan Hospital, FuDan University, Shanghai, China; ^5^ Department of General Surgery, The Second Hospital of Dalian Medical University, Dalian, China; ^6^ Department of Pathology, Changhai Hospital, Navy Medical University, Shanghai, China; ^7^ Department of Breast and Thyroid Surgery, Changhai Hospital, Navy Medical University, Shanghai, China

**Keywords:** NCAPG, glioma, immune infiltrates, NK cells, immunohistochemistry

## Abstract

Glioma is one of the most deadly types of brain cancer. As it is highly invasive, the prognosis for glioma patients remains dismal, with median survival rarely exceeding 16 months. Thus, developing a new prognostic biomarker for glioma and investigating its molecular mechanisms is necessary for the development of an efficient treatment strategy. In this study, we analyzed a cohort of 1,131 glioma patients using RNA-seq data from The Cancer Genome Atlas (TCGA project) and Gene Expression Omnibus (GSE4290 and GSE16011 datasets), and validated the results using the RNA-seq data of 1,018 gliomas from the Chinese Glioma Genome Atlas (CGGA project). We used the R language as the main tool for statistical analysis and data visualization. We found that NCAPG, a mitosis-associated chromosomal condensing protein, is highly expressed in glioma tissues. Furthermore, the expression of NCAPG increased significantly with the increase in tumor grade, and high NCAPG expression was found to be a predictor of poor overall survival in glioma patients (*P* < 0.001). This result shows that NCAPG expression could be an independent prognostic factor. Importantly, when the expression of NCAPG was knocked down, the CCK-8 assay revealed that the proliferation of glioma cells (LN-229 and T98G cell lines) decreased significantly compared with the control group. In addition, the healing rates of these cells were significantly lower in the si-NCAPG group than in the control group (*P* < 0.001). We then used the CIBERSORT algorithm to analyze the expression levels of 22 subpopulations of immune cells and found that NCAPG was significantly negatively correlated with natural killer cell activation. In addition, it was positively correlated with MHC-I molecules and ADAM17. Our study is first in comprehensively describing the high expression of NCAPG in glioma. It also shows that NCAPG can function as an independent prognostic predictor of glioma, and that targeting NCAPG can be a new strategy for the treatment of glioma patients.

## Introduction

Glioma is the most common and aggressive primary malignant tumor of the central nervous system, accounting for 50–60% of intracranial tumors (1, 2). At present, combination therapy, including surgery, radiotherapy, and chemotherapy, is the standard treatment strategy for glioma, but its prognosis is still poor because of the difficulty of complete resection and its low sensitivity to radiotherapy and chemotherapy (3–5). Glioma is usually associated with poor prognosis, with a 5-year survival rate of only 10–20% (6, 7). In recent years, with the development of molecular pathology, some molecular markers in glioma have played an important role in the diagnosis and prognosis of the disease, such as isocitrate dehydrogenase (IDH), epidermal growth factor receptor (EGFR), O-6-methylguanine-DNA methyltransferase (MGMT), and tumor protein p53 (TP53) (8). In 2016, the WHO Classification of Tumors of the Central Nervous System (WHO CNS4) was first to use molecular markers in glioma classification, and in 2021 WHO CNS5 placed even more emphasis on their importance (9, 10). Therefore, the new molecular classification of glioma may play a key role in its prognosis.

With the rise of tumor immunotherapy, the study of the glioma immune microenvironment has gradually become a research hotspot, and rapid progress has been made in this field (11, 12). However, the effect of clinical treatment is still not ideal, and the cure and survival rates of glioma patients have not improved significantly in the past ten years, which may be due to the immune escape caused by the tumor immune microenvironment (TME). It is well known that the TME contains many different non-cancerous cell types in addition to cancer cells, including endothelial cells, pericytes, fibroblasts, and immune cells (13). Immune cells within the TME play an important role in tumorigenesis. Over the past few decades, it has been commonly accepted that the brain is “immunologically privileged” owing to the separation of the central nervous system (CNS) from the systemic immune system because of the blood brain barrier (BBB). However, the traditional view is gradually being challenged owing to advances in the field of brain cancer research. It was reported that the BBB is often compromised in certain brain tumors, causing a robust infiltration of multiple immune cell types from the peripheral circulation (14). Thus, focusing on the glioma immune microenvironment may help to provide new ideas for its treatment.

Non-structural maintenance chromatin condensin 1 complex subunit G (NCAPG) is a mitosis-associated chromosomal condensing protein widely present in eukaryotic cells (15, 16). NCAPG is a polypeptide composed of 1015 amino acids with a relative molecular weight of 114.1 kDa (17, 18). It is highly expressed in a variety of cancers such as prostate cancer, breast cancer, and hepatocellular carcinoma (HCC) (19–22), and has been reported to be associated with invasion, metastasis, apoptosis, and drug resistance of tumor cells through various molecular mechanisms (23). However, whether NCAPG can be used as a biomarker of glioma and its function in gliomas have not yet been reported.

In the present study, we found that NCAPG was significantly overexpressed in glioma by analyzing the RNA-seq datasets of 698 gliomas obtained from The Cancer Genome Atlas (TCGA) network and 433 gliomas obtained from the Gene Expression Omnibus (GSE4290 and GSE16011) database. We validated the results using the Chinese Glioma Genome Atlas (CGGA) database and determined that the NCAPG gene could be further investigated as an independent prognostic factor for glioma. In addition, overexpression of NCAPG was found to promote the proliferation, migration, and differentiation of glioma cells. Furthermore, we found that MHC-I was overexpressed when NCAPG was overexpressed, resulting in immune escape from natural killer (NK) cells. This study aimed to evaluate NCAPG expression in glioma tissues and investigate the role of NCAPG in the development and prognosis of glioma.

## Materials and Methods

### Microarray Data Information

The data used in our study were obtained from the public databases TCGA, NCBI-GEO, and CGGA. The TCGA and NCBI-GEO data were used as the experimental groups and the CGGA data were used as the validation set. The expression data of TCGA-glioma, including TCGA- low-grade tissues (LGG) and TCGA- high-grade glioma tissues (GBM), from the Affymetrix HT Human Genome U133a microarray platform, were downloaded from the TCGA (https://portal.gdc.cancer.gov/) database. The dataset consisted of the clinical data of 698 glioma patients and 5 normal individuals, and patients with incomplete clinical data were excluded from subsequent analyses. In addition, we downloaded two GEO datasets (GSE16011 and GSE4290) from the Affymetrix Human Genome U133 Plus 2.0 Array platform, containing the clinical data of 433 glioma patients and 31 normal individuals. The validation set (CGGA data), gene expression data, and corresponding clinical data of the glioma patients were downloaded from CGGA (LGG+GBM) (http://www.cgga.org.cn/). Two datasets containing the clinical data of 693 and 325 patients (Dataset ID: mRNAseq_693 and mRNAseq_325, Data Type: RNA sequencing), respectively, were downloaded. The two sets of gene expression data were corrected in batches and integrated by loading them into the Limma (14) and SVA (15) packages in the R software (R version 4.0.4; https://www.r-project.org/). As in the case of TCGA data, the patients with incomplete clinical data were excluded from subsequent analyses.

### Data Processing of Differentially Expressed Genes (DEGs)

The DEGs in GSE16011 and GSE4290 between glioma and normal specimens were identified using GEO2R online tools with the criteria |logFC| > 2 and adjusted *P* value < 0.05. For the TCGA glioma dataset, we used the limma package of R to analyze the differences between the tumor and normal tissues using the criteria |logFC| > 2 and adjusted *P* value < 0.05. Subsequently, the raw data in TXT format were checked using the Venn software (Draw Venn Diagram; http://bioinformatics.psb.ugent.be/webtools/Venn/) to detect the common DEGs among the three datasets. The DEGs with logFC < 0 were considered as downregulated genes, while those with logFC > 0 were considered as upregulated.

### PPI (Protein–Protein Interaction) Network and Gene Enrichment Analysis

The PPI network was predicted using the online search tool for the retrieval of interacting genes (STRING; http://string-db.org) database. A sufficient understanding of the functional interaction between proteins can provide better insight into the underlying mechanisms causing the initiation or development of cancers. Therefore, we constructed a PPI network based on the STRING database, using the Cytoscape software (National Institute of General Medical Sciences, USA). Kyoto Encyclopedia of Genes and Genomes (KEGG) and Gene Ontology (GO) analyses were performed using the DAVID database (DAVID; http://david.ncifcrf.gov). Gene set enrichment analysis (GSEA) (version 4.1.0, the broad institute of MIT and Harvard, http://software.Broadinstitute.org/gsea/downloads.jsp) of glioma and adjacent normal tissues was performed to investigate the biological characteristics of glioma.

### Survival Prognostication

The overall survival (OS) was calculated from the date of initial diagnosis to the date of the last follow-up or death, using the survival data obtained from the CGGA and TCGA databases. All samples were divided into two expression groups (high and low) according to the median value of NCAPG expression. The prognostic value of NCAPG in these cohorts was evaluated using the Kaplan–Meier method and the log-rank test.

### Cell Culture

The LN-229 cell line was derived from the right frontal parieto-occipital cortex of a 60-year-old female patient with glioblastoma. The T98G cell line was derived from the brain of a 61-year-old male patient with glioblastoma multiforme. These cell lines were obtained from the Institute of Pathology and Southwest Cancer Center of Army Medical University (Chongqing, China) and cultured in Dulbecco’s modified Eagle medium (DMEM; Gibco, Carlsbad, CA, USA) supplemented with 1% penicillin/streptomycin (Beyotime, China) and 10% fetal bovine serum (Gibco) at 37°C.

### Verification of the Role of NCAPG in Glioma Cells

NCAPG siRNA (si-NCAPG-1, -2, and -3) and negative control (si-NC) were purchased from Tsingke (Shanghai, China); their sequences are listed in **Table S1**. Lipofectamine RNAiMAX (Invitrogen, Carlsbad, CA, USA) was used for transfection in accordance with the manufacturer’s instructions. Cell viability at different time points was determined using the CCK-8 assay (GLPBIO, China) and the healing rates were evaluated by cell scratch test.

### Quantitative Real Time PCR (RT-qPCR)

Total RNA was isolated from glioma tissues and cell lines using TRIzol RNA isolation reagent (Invitrogen) and reverse transcribed to cDNA using a cDNA Synthesis kit (Takara, Shiga, Japan). The gene expression levels were detected by RT-qPCR using SYBR Premix Ex Taq (Takara). The primers used are listed in **Table S2**.

### Western Blot Analysis

Proteins were isolated from cultured cells and tissues using SDS lysis buffer with freshly added protease inhibitor. Equal amounts of proteins were separated using 8% SDS-PAGE and transferred to polyvinylidene fluoride membranes (Millipore, Billerica, MA, USA). The membranes were incubated with antibodies against NCAPG (PA5-101540, 1:1000; Invitrogen) (1:2500, Proteintech, Hubei, China), MHC class I (ab134189, 1:5000; Abcam, Cambridge, MA, USA) and β-actin (1:1000; Abcam). Chemiluminescence was detected using a High-sig ECL Western Blotting Substrate (Tanon, Shanghai, China) detection system.

### Transwell Invasion Assay

The cell invasion assay was performed using a 24-well Transwell chamber (Corning Incorporated, Corning, NY, USA). Cells were seeded at a density of 2 × 10^4^ cells in the upper chamber (pore size, 8 µm), which was precoated with Matrigel (BD Biosciences, Franklin Lakes, NJ, USA). The lower chamber was filled with 600 µL DMEM containing 10% FBS. Following a 36-h incubation at 37°C, cells on the upper-side of the membrane were removed using clean swabs. Invaded cells on the lower surface of the membrane were fixed with 4% paraformaldehyde. Then, cells were stained with gentian violet. Invaded cells in three fields were counted under a microscope.

### CIBERSORT

The TCGA-glioma database was analyzed using the CIBERSORT software (https://cibersort.stanford.edu). Twenty-two types of immune cells were evaluated to estimate the correlation between NCAPG and infiltrating immune cells.

### Immunohistochemical Staining

To analyze the expression of NCAPG in glioma, we purchased glioma tissue microarrays (TMA) from the Outdo Biotech Company (Shanghai, China). The TMA included 140 primary glioma tissues that had been operated on and had pathologic data from 2008 to 2011. The date of the operation was used as the initial time for follow-up. Regular follow-up visits were performed annually for the included patients. The TMA slides were subjected to immunohistochemical (IHC) analysis using the Envision method. A total of 140 glioma tumor samples were stained with NCAPG antibody (ab251864, 1:1000; Abcam). Two independent pathologists evaluated the expression of NCAPG in glioma tissues in a double-blind manner. The IHC score was calculated as follows (24): total score = intensity score × percentage score. The intensity score was measured based on the staining intensity and was classified into four levels: negative (0), weak (1), moderate (2), and strong (3). The percentage score was based on the percentage of positive cells in staining and was classified into five levels: < 5% (0), 5–25% (1), 25–50% (2), 50–75% (3), and > 75% (4). Finally, the total IHC score, the maximum of which is 12 points, was classified into four levels: negative (0), weak positive (1–4), positive (5–8), and strong positive (9–12). For subsequent experiments, the negative and weak positive samples were categorized as belonging to the low expression group, while the positive and strong positive samples were categorized as belonging to the high expression group.

### Statistical Analysis

Statistical data acquired from TCGA and CGGA were merged and analyzed using R 4.0.3. Receiver operating characteristic (ROC) curves were used to establish the diagnostic value of NCAPG in gliomas. The area under the curve (AUC) and *P*-values were calculated. Correlations between clinical information and NCAPG expression were analyzed using logistic regression. Furthermore, multivariate Cox analysis was used to evaluate the influence of NCAPG expression and other clinicopathological factors on survival. To detect the correlation between immune cells, we created a correlation heatmap based on the correlation between every two different immune cells in the samples. Statistical analyses were performed using the SPSS (ver. 22.0, IBM Corp., Armonk, NY, USA) and GraphPad Prism 8 software (GraphPad Software Inc., USA). Student’s t-test was used to analyze the differences between two groups, while one-way analysis of variance was used to analyze more than two groups. The *χ^2^* test was used to evaluate the differences in nuclear NCAPG expression in glioma tissues. *P*-value < 0.05 was set as the cut-off criterion (*P < 0.05, **P < 0.01, and ***P < 0.001).

## Results

### Identification of DEGs in Glioma

**Figure 1:**
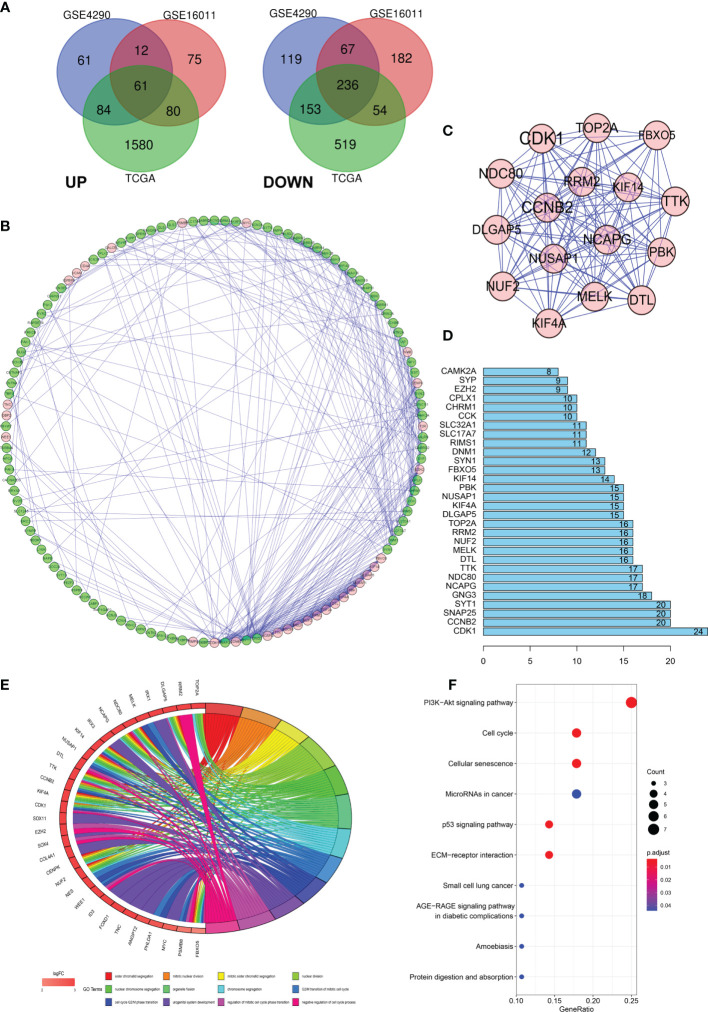
Differentially expressed genes (DEGs) in glioma and the functional analysis of DEGs. **(A)** Venn diagram of DEGs based on TCGA and GEO. **(B–D)** Protein–protein interaction (PPI) of DEGs. **(E, F)** Gene ontology (GO) and pathway enrichment analysis of the glioma enriched DEGs.

Data from 1131 glioma and 36 normal tissue samples were analyzed in the present study. Using GEO2R online tools and the R software, we extracted the DEGs from GSE4290, GSE16011, and TCGA-glioma datasets. We then used the Venn diagram software and identified a total of 297 DEGs that were common to the three datasets, including 236 downregulated (logFC < 2) and 61 upregulated genes (logFC > 2) in the glioma tissues compared with normal tissues (**Figure 1A**).

### PPI Network and GO Enrichment and KEGG Analyses

To further explore the underlying mechanisms, we constructed a PPI network of the DEGs based on the STRING database (http://string-db.org) using the Cytoscape software. The interactions between the 297 genes are shown in **Figure 1B**, and the significance of the module was screened using MCODE. CDK1, CCNB2, NCAPG, NCD80, TTK, and DTL were the hub nodes with the highest node degrees in the module (**Figure 1C**). In addition, the bar plots represent the top 30 genes ranked by the number of nodes (**Figure 1D**). To obtain an in-depth understanding of these upregulated DEGs, GO term enrichment and KEGG pathway analyses were performed using DAVID software (**Figures 1E, F**).

### Expression Level of NCAPG in Glioma Patients and GSEA

**Figure 2:**
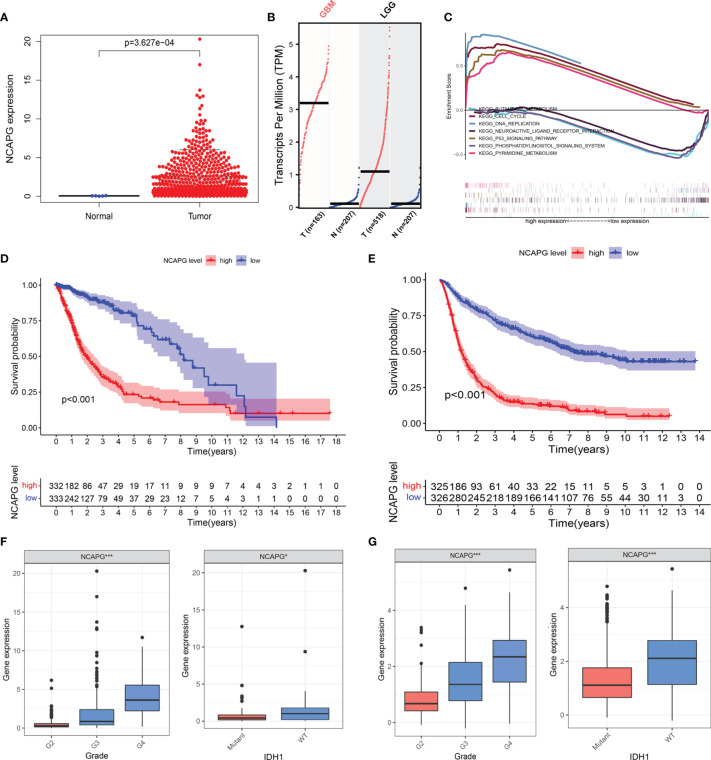
NCAPG is highly expressed in glioma tissues and could be a biomarker for glioma. **(A)** NCAPG is highly expressed in glioma tissue compared with normal tissue. **(B)** The expression of NCAPG in high-grade glioma tissues (GBM) and low-grade glioma tissues (LGG) analyzed by GEPIA. **(C)** Significantly enriched pathways associated with NCAPG by gene set enrichment analysis (GSEA). **(D, E)** Survival curve of NCAPG expression in TCGA and CGGA, respectively. **(F, G)** Differential expression of NCAPG in different cancer grades and isocitrate dehydrogenase (IDH) mutations. (*P < 0.05, ***P < 0.001).

The clinical and gene expression data were obtained from TCGA. We analyzed the differential expression of NCAPG in glioma and normal tissues using TCGA (**Figure 2A**) and then used the GEPIA online analysis website (http://gepia.cancer-pku.cn/index.html) for the verification of the results (**Figure 2B**). The results showed that NCAPG was highly expressed in glioma tissues, while it was almost absent in normal tissues. To further understand the cellular roles of NCAPG in glioma, GSEA was performed. The top four normalized enrichment scores (NES) of the groups with high NCAPG expression were of the KEGG cell cycle (NES = 2.28), KEGG p53 signaling pathway (NES = 2.26), KEGG pyrimidine metabolism (NES = 2.17), and KEGG DNA replication (NES = 2.00) (**Figure 2C**).

### NCAPG Expression Is Significantly Correlated With the Degree of Malignancy and Glioma Subtype

To further verify the role of NCAPG in the progression of glioma, we used the TCGA dataset to explore the relationship between the expression level of the NCAPG gene, patient survival, tumor classification, and isocitrate dehydrogenase (IDH) mutations. At the same time, we used the CGGA database to further expand the experimental sample size and verify the study content in order to avoid possible deviations caused by a single database. The results of the Kaplan–Meier analysis indicated that the patients with high NCAPG expression had a shorter survival compared with those presenting low NCAPG expression, in both the TCGA and CGGA datasets (**Figures 2D, E**; *P* < 0.001). Furthermore, we also found that the expression level of NCAPG was more or less related to the grade of the glioma, and that high NCAPG expression usually corresponded to high-grade tumor samples. The expression level of NCAPG in GBM of WHO grade 4 was significantly higher than that of other pathological subtypes (**Figure S1**) using the GlioVIS online analysis website (http://gliovis.bioinfo.cnio.es). In addition, the expression level of NCAPG was also related to the mutation of IDH, with the wild-type IDH group exhibiting higher levels of NCAPG compared with the IDH mutation group (**Figures 2F, G**).

### Univariate and Multivariate Analysis Showed the Prognostic Significance of NCAPG and Its Association With Related Clinicopathological Factors in Glioma

**Figure 3:**
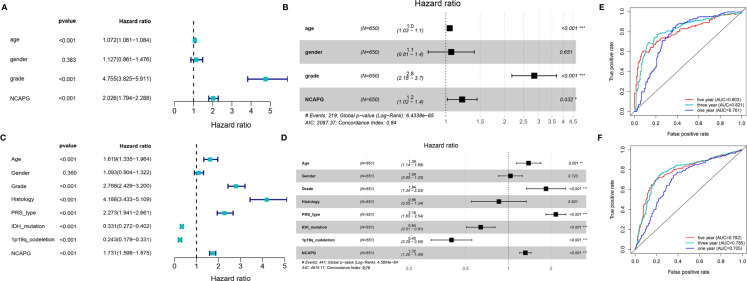
Construction and verification of single gene diagnostic model. **(A, B)** Univariate and multivariate COX analysis of NCAPG using the TCGA database. **(C)** The predictive capacity of the risk score for the 1- year, 3- year, and 5- year survival rates of patients in TCGA; AUC, area under the curve. **(D, E)** Univariate and multivariate COX analysis of NCAPG using the CGGA database. **(F)** The predictive capacity of the risk score for the 1- year, 3- year, and 5- year survival rates of patients in CGGA; AUC, area under the curve. (*P < 0.05, **P < 0.01, ***P < 0.001).

To further explore the function of NCAPG and determine whether the risk score was an independent and significant prognostic factor in glioma, we performed univariate and multivariate Cox regression analyses in the above datasets. We discovered that NCAPG may serve as an independent risk factor in the TCGA-glioma dataset (**Figures 3A, B**). We then used the NCAPG single-gene model to predict patient survival; the AUC of the risk score for the 5-year, 3-year, and 1-year survival prediction was 0.803, 0.821, and 0.761 (**Figure 3C**), respectively. We also analyzed NCAPG expression and related clinical features in the CGGA database and found consistent trends (**Figures 3D, E**). The AUC of the risk score for the 5-year, 3-year, and 1-year survival predictions were 0.782, 0.785, and 0.705, respectively (**Figure 3F**). In summary, NCAPG may act as an independent prognostic factor for gliomas.

### NCAPG Promotes Glioma Proliferation, Migration, and Differentiation

**Figure 4:**
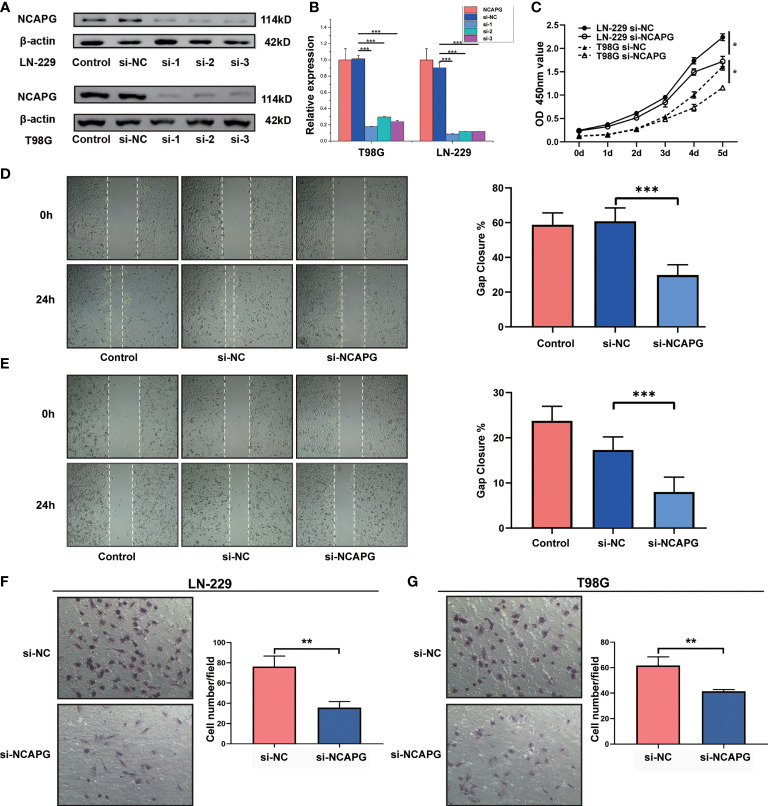
NCAPG promotes the proliferation, migration, and differentiation of glioma. **(A)** NCAPG expression in LN-229 and T98G cell lines were analyzed using western blot after transfection with si-NC, si-1, si-2, and si-3. **(B)** Quantitative analysis of the expression of NCAPG in LN-229 and T98G cell lines by western blot and RT-qPCR. **(C)** Cell viability of LN-229 and T98G cell lines at different time points after incubation, analyzed by CCK-8. **(D, E)** Cell scratch test was used to detect the healing rates of LN-229 and T98G cell lines. **(F, G)** Transwell invasion assay was used to detect the invasion ability of both LN-229 and T98G cell lines. (*P < 0.05, **P < 0.01, ***P < 0.001).

To demonstrate the role of NCAPG in glioma, glioma cell lines (LN-229 and T98G) were transfected to silence the NCAPG gene. The transfected glioma cell lines were categorized into control group (Control), silenced negative control group (si-NC), silenced interference sequence 1 (si-1), silenced interference sequence 2 (si-2), and silenced interference sequence 3 (si-3). We then analyzed the expression of NCAPG in the transfected cell lines using western blotting and RT-qPCR. As shown in **Figures 4A, B**, the expression levels of NCAPG in the si-1, si-2, and si-3 groups were lower than those in the Control and si-NC groups; in particular, the si-1 group showed the lowest expression levels among the si-1, si-2, and si-3 groups; therefore, we chose the si-1 group (hereafter denoted as si-NCAPG) to use in subsequent experiments.

Furthermore, the CCK-8 assay revealed the effect of NCAPG on glioma cell proliferation. As shown in **Figure 4C**, NCAPG silencing (si-NCAPG group) significantly decreased the proliferation of glioma cells (LN-229 and T98G) in contrast with the respective si-NC groups. In addition, the healing rates of glioma cells (LN-229 and T98G), detected by the cell scratch test, were significantly lower in the si-NCAPG group than in the control group (**Figures 4D, E**, *P* < 0.001). Transwell invasion assays were conducted to evaluate the effects of NCAPG silencing on the invasive abilities of glioma cells (LN-229 and T98G). The results demonstrated that the ability of glioma cells to invade through the Matrigel matrix was significantly decreased in the si-NCAPG group compared with the si-NC group (**Figures 4F, G**).

### High NCAPG Expression Is Associated With High Grade and Poor Prognosis in Glioma

**Figure 5:**
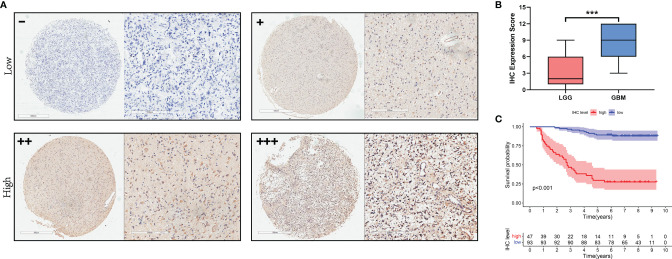
The expression of NCAPG in glioma TMA. **(A)** The expression and distribution of NCAPG in representative tissue specimens. **(B)** The relationship between NCAPG expression and glioma grade in the tissue microarrays (TMA). **(C)** Survival curve and NCAPG expression in glioma TMA. (-: Negative for a score of 0, +: Weak for a score of 1-4, ++: Moderate for a score of 5-8, +++: Positive for a score of 9-12, ***P < 0.001).

To investigate the relationship between NCAPG and glioma tissues, IHC staining was performed on TMA slides. We collected 140 glioma tissue samples; the main clinicopathological variables examined in this study are shown in **Table 1**. Then, we divided the samples into two expression groups, high and low, according to the immunohistochemical score, and their clinical and survival data were analyzed. As shown in **Figure 5A**, NCAPG was located in the cytoplasm, and the χ^2^ test showed that the expression of NCAPG in GBM (WHO 4) was significantly higher than that in LGG (WHO 1–3) (*P*<0.001) (**Table 1**), suggesting that NCAPG expression was positively correlated with histological grade (**Figure 5B**). At the same time, we also found that the expression level of NCAPG in tumor tissues was higher in older patients than in younger patients. However, the expression level of NCAPG in glioma was not correlated with the sex of the patient or the expression levels of Ki-67 and GFAP (**Table 1**). The survival curve is shown in **Figure 5C**, which indicates that the survival rate of patients in the high expression group was significantly lower than that in the low expression group. In summary, NCAPG can determine the prognosis and act as a new biomarker for glioma.

**Table 1:** NCAPG expression and clinicopathological parameters in glioma tissue chips.

Clinicopathological parameters	Total Cases (n)	IHC Staining of NCAPG				*χ^2^*	*P*
		Low expression		High expression			
		Score 0	Score 1–4	Score 5–8	Score 9–12		
Age(years)							
≤40	62	11	38	10	3	7.927	0.007*
>40	78	5	39	18	16		
Sex							
Male	96	9	53	18	16	0.466	0.566
Female	44	7	24	10	3		
Histological grade (WHO)							
WHO 1	17	16	1	0	0	51.944	<0.001*
WHO 2	64	7	49	8	0		
WHO 3	40	2	16	13	9		
WHO 4	19	0	3	6	10		
Recurrence							
With	71	4	42	15	10	0.174	0.723
Without	69	12	35	13	9		
Ki-67 (IHC)							
<5%	69	10	39	14	6	2.6	0.452
5%–10%	44	4	27	8	5		
10%–20%	12	1	5	5	1		
>20%	15	1	8	3	3		
GFAP (IHC)							
Low (+)	66	8	38	11	9	0.822	0.663
Middle (++)	65	6	36	18	5		
High (+++)	9	2	5	1	1		

Significant P-values are shown in italics, *P < 0.05.

### Relationship Between NCAPG Expression and Tumor-Infiltrating Immune Cells

**Figure 6:**
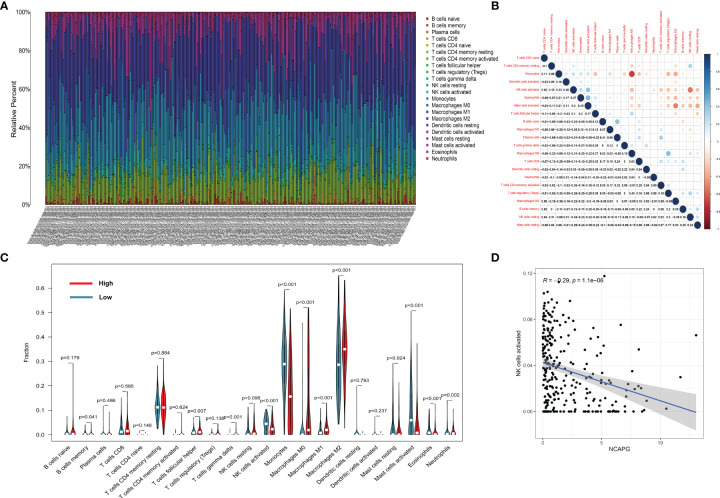
The landscape of immune infiltration in glioma cells. **(A)** The proportions of immune cells in each glioma sample are indicated with different colors, and the lengths of the bars in the bar chart indicate the levels of the immune cell populations. **(B)** Correlation matrix for all 22 immune cell proportions. Some immune cells were negatively related, represented in blue, while others were positively related, represented in red. The darker the color, the higher the correlation (*P* < 0.05). **(C)** Violin plot visualizing the differentially infiltrated immune cells. **(D)** the relation between the expression of NCAPG and NK cell activation.

To display the distribution of immune infiltration in glioma, we first explored the immune infiltration of 22 subpopulations of immune cells in glioma tissue using the CIBERSORT algorithm. As shown in **Figure 6A**, the divergence in tumor-infiltrating immune cells (TIICs) may be a distinctive feature of individual differences and have prognostic value.

**Figure 6B** shows that the populations with a significantly negative relationship included M0 macrophages and monocytes (−0.69), activated and resting NK cells (−0.54), and M0 macrophages and activated mast cells (−0.5). The populations with a significantly positive relationship were eosinophils and activated mast cells (0.45), M0 macrophages and Tregs cells (0.43), plasma cells and naïve B cells (0.41), and activated NK cells and activated mast cells (0.40).

The results of the difference and correlation analyses showed that a total of 11 TIICs were correlated with the expression of NCAPG (**Figure 6C**). Among them, five types of TIICs were positively correlated with NCAPG expression, including macrophage M0, macrophage M1, macrophage M2, neutrophils, and resting NK cells, while six types were negatively correlated with NCAPG expression, including memory B cells, activated dendritic cells, eosinophils, activated mast cells, monocytes, and activated NK cells. These results further support that the levels of NCAPG affect the immune activity of the TME. **Figure 6D** shows that NCAPG has a significantly negative relationship with NK cell activation.

### NCAPG Affects NK Cell Activation

**Figure 7:**
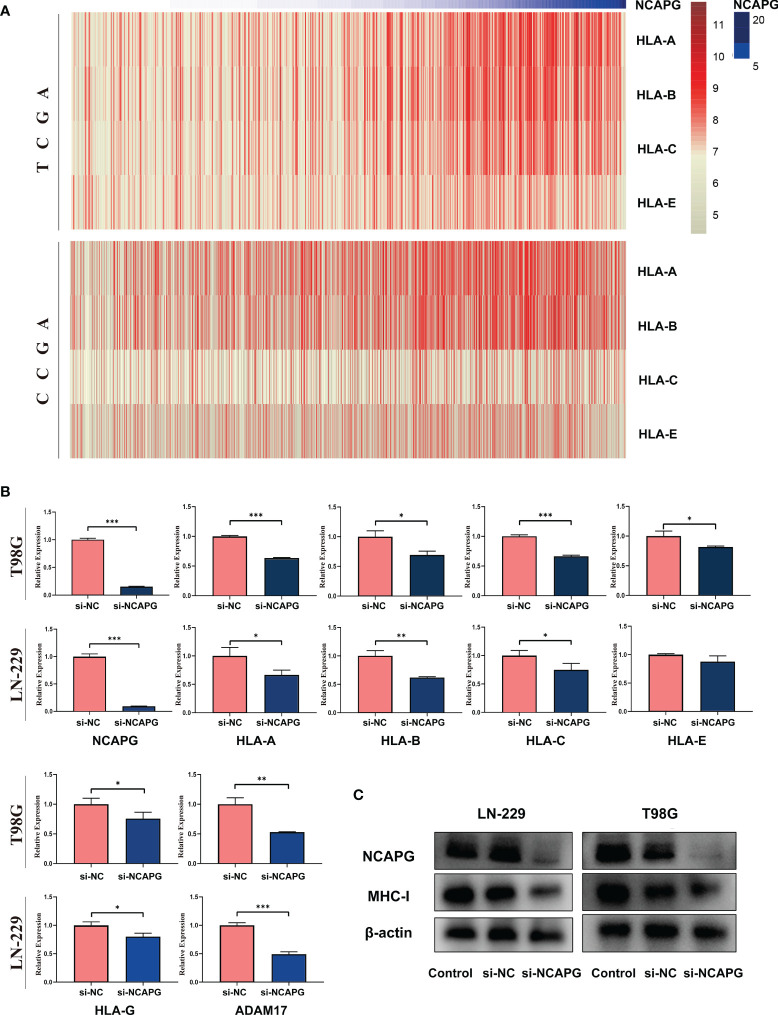
NCAPG affects the expression of major histocompatibility complex-I (MHC-I) in glioma. **(A)** Heatmap of the relationship between NCAPG expression and MHC-I in the TCGA and CGGA datasets. **(B)** The expression of NCAPG in relation to different MHC-I molecules and ADAM17 with RT-qPCR assay. **(C)** The relationship between NCAPG and MHC-I molecule expression using western blot analysis. (*P < 0.05, **P < 0.01, ***P < 0.001).

Since we found a negative relationship between the expression of NCAPG and NK cells activation, we explored the mechanism underlying this effect. First, we analyzed the relationship between the expression of NCAPG and major histocompatibility complex-I (MHC-I) using the TCGA and CGGA glioma datasets. As shown in **Figure 7A**, the heatmap indicated that the expression of NCAPG was positively correlated with MHC-I molecules, especially HLA-A, HLA-B, and HLA-C. Next, we verified the results using the T98G and LN-229 glioma cell lines and found that the interference efficiency of si-NCAPG in both cell lines was as high as 80%; **Figures 7B, C** show that the expression level of MHC-I molecules decreased by varying degrees according to the decrease in NCAPG expression, especially HLA-A, HLA-B and HLA-C, which is consistent with the results obtained from the TCGA and CGGA datasets. Furthermore, we tested the expression of α-disintegrin and metalloprotease-17 (ADAM17) in glioma tissues and found that the expression of ADAM17 decreased with the reduction in NCAPG expression.

## Discussion

NCAPG, a protein component of the condensation complex, is important for chromosomal stabilization and condensation during meiotic and mitotic cell division (25). NCAPG has been shown to be relevant in a number of different cancer types, including liver cancer (26), bladder cancer (27), renal cell carcinoma (28), multiple myeloma (29), melanoma (30) and breast cancer (31). However, the exact role of NCAPG in glioma, and more generally in cancer, remains to be determined.

Therefore, this study explored the relationship between the expression of NCAPG and glioma, illustrated the relationship between the expression of NCAPG and the glioma immune microenvironment, and explored the mechanism by which NCAPG affects NK cell activation in glioma. First, we comprehensively analyzed the TCGA-glioma and GEO (GSE 10611 and 4290) datasets and detected a high expression of NCAPG in glioma; we then verified the results using the GEPIA online website. The functional enrichment of differentially expressed genes and NCAPG in gliomas predicted that NCAPG may be closely related to the cell cycle (32), p53 signaling pathway (33), and PI3K/ARK signaling pathway (34) in glioma, which is consistent with previous studies on NCAPG in tumors. Furthermore, we analyzed the relationship between the expression of NCAPG in glioma and the survival time of glioma patients, tumor classification, and IDH mutation using TCGA data. The results showed that high expression of NCAPG could decrease the survival rate of patients. The results also highlighted that, in glioma patients, the expression of NCAPG is associated with the tumor grade and that NCAPG expression increases significantly with the tumor grade. The expression of NCAPG in GBM was significantly higher than that in other pathological gliomas. Moreover, glioma patients with wild-type IDH exhibited an increased expression of NCAPG. In addition, using Cox analysis, we identified that the expression level of NCAPG in glioma can serve as an independent prognostic factor in glioma patients. To avoid bias in the analysis caused by a single database (TCGA), we used the CGGA database to increase the number of data sources and validate the results obtained, and found consistency in the results. In addition, we used glioma TMA to perform IHC analysis. The results showed the significance of NCAPG expression in determining glioma prognosis and tumor grade.

Next, we explored the relationship between the expression level of NCAPG and the biological properties of glioma using LN-229 and T98G cell lines. We decreased the expression level of NCAPG within the cell lines using si-NCAPG and confirmed the knockdown efficiency using RT-qPCR and western blotting. The knockdown of NCAPG significantly decreased cell proliferation, migration, and invasion ability of glioma cells. This result is consistent with those of other studies which evaluated the function of NCAPG in other tumors; for example, Zhang et al. (35) reported that NCAPG could induce cell proliferation in cardia adenocarcinoma *via* the PI3K/AKT signaling pathway, and Xu et al. (32) revealed that elevated mRNA expression of NCAPG is associated with poor prognosis in ovarian cancer. In conclusion, our results suggest that NCAPG expression is associated with malignant biological processes and can be used as a biomarker for glioma. NCAPG expression in glioma is likely to be key in diagnostic and treatment strategies for glioma.

Nowadays, immunotherapy for tumors has become a new hotspot, and checkpoint blocking antibodies, such as anti-PD1/PD-L1, are being widely used in the treatment of multiple solid tumors. The relationship between NCAPG expression in tumors and the immune microenvironment has not yet been explored. We conducted a correlation analysis between the expression levels of NCAPG in tumors and 22 types of immune cells in the immune microenvironment using the TCGA-glioma data set, and found a negative relationship between the expression of NCAPG and NK cell activation, which attracted our attention.

NK cells, an important type of immune cell in the TME, have been reported to recognize and kill infected and abnormal cells without prior stimulation (36). Therefore, NK cells play a key role in immune surveillance of viral infectious diseases and cancers. It has been reported that the killing effect of NK cells is regulated by the signaling between the activating and inhibiting receptors on their surface (37). There are two types of receptors on the surface of NK cells: activating receptors, including NKG2D, NKp30, NKp46, and CD16, and inhibitory receptors, including KIR2DL1, KIREDL2/3, and KIR3DL1 (38, 39). Activating receptors such as NKG2D kill target cells by identifying and binding to their specific ligands, such as MICA, MICB, or ULBP proteins; however, inhibitory receptors can prevent the killing of target cells by identifying and binding to their specific ligands, such as MHC-I molecules, to produce the inhibiting signal.

Based on the TCGA and CGGA data of glioma tissues, we found that the expression of MHC-I molecules increased with the increase in NCAPG expression. Subsequently, we identified a positive correlation between the expression of NCAPG and MHC-I molecules in the glioma cell lines (LN-229 and T98G). When NCAPG was knocked down by si-NCAPG in these cell lines, the expression of MHC-I molecules also decreased to a certain extent. It is well known that when MHC-I molecules on the cancer cell surface are reduced or even absent, NK cells respond to the missing self of MHC-I molecules by releasing soluble particles to produce a killing effect. In addition, our study demonstrated that the expression of ADAM17 in the glioma cell lines was positively correlated with the expression of NCAPG. The ADAM17 expressed on the surface of tumor cells has been reported to bind to the activating receptors (CD16 and NKG2D) of NK cells and rupture them (40); at the same time, it acts as a proteolytic enzyme by participating in the shedding of B7-H6, which is highly expressed in tumor cells (41, 42). The presence of the soluble form of B7-H6 has also been associated with lower levels of activating receptor NKp30 expression on NK cells in cancer. Overall, we can conclude that the expression of NCAPG in glioma is relatively high, and that the expression of MHC-I molecules and ADAM17 in glioma cells is also elevated, thereby inhibiting the activation of NK cells, resulting in immune escape and decreased patient survival.

**Figure 8:**
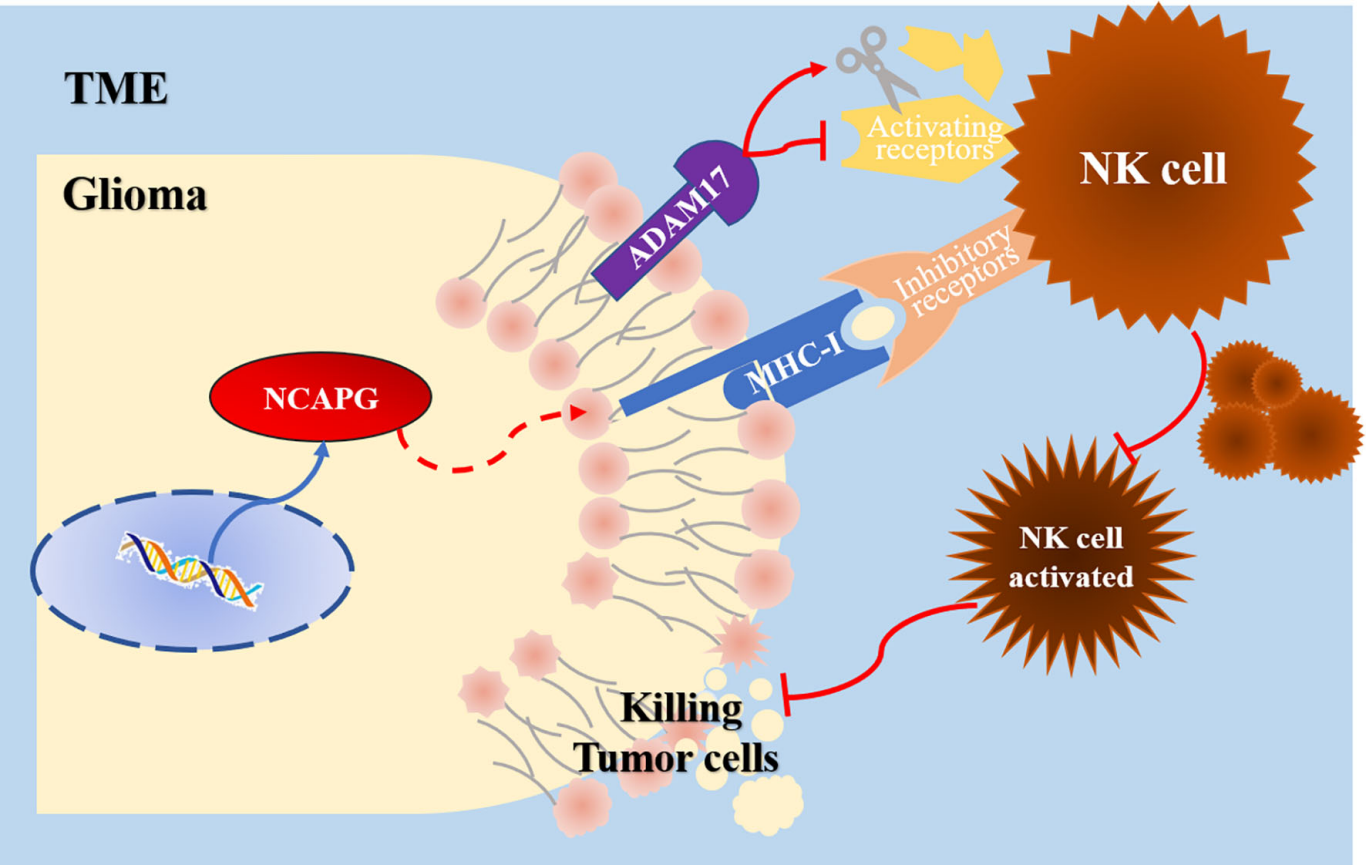
The potential mechanism of NCAPG function in glioma TME tumor immune cell infiltration.

In conclusion, this study revealed the high expression of NCAPG in glioma tissues through comprehensive RNA-seq analysis. The expression level of NCAPG in glioma can reflect, to a certain extent, the tumor grade and prognosis of patients. High expression levels of NCAPG were significantly correlated with poor survival in glioma patients, and could also enhance the proliferation, migration, and invasion ability of glioma cells. In addition, Cox regression analysis showed that NCAPG can act as an independent prognostic factor and as a tumor marker for glioma. Most importantly, we confirmed the negative correlation between NCAPG and the activation of NK cells through CIBERSORT analysis, and explored the possibility of immune escape from NK cell cytotoxicity through RT-qPCR and western blot. These results indicate that high expression of NCAPG in glioma can increase the expression of MHC-I and AMAD17 molecules on the tumor surface, thus camouflaging the tumor and preventing NK cells from being activated in the immune microenvironment. Therefore, NK cells lose their monitoring and killing effect on the tumor, resulting in an acceleration of its proliferation and invasion (**Figure 8**). Therefore, further studies on NCAPG will be helpful for the diagnosis and treatment of glioma in the future. However, this study did not involve *in vivo* experiments, which will be included in a follow-up study to explore in depth the specific mechanism of NCAPG regulation of the immune microenvironment, and attempt to use NCAPG as a target for glioma treatment to evaluate its therapeutic effect.

## Data Availability Statement

The original contributions presented in the study are included in the article/**Supplementary Material**, further inquiries can be directed to the corresponding authors.

## Ethics Statement

The studies involving human participants were reviewed and approved by Research Ethics Committee of the Second Affiliated Hospital of Dalian Medical University, Dalian, China. Written informed consent for participation was not required for this study in accordance with the national legislation and the institutional requirements.

## Author Contributions

HW and HL conceived and designed the study. GZ and TH collected and analyzed the data. GZ completed the experimental cell manipulation. ZY organized the data of CGGA and TGGA. JW completed immunohistochemistry of TMA and collected patient clinical data. XH and JW completed the immunohistochemical scoring. ZY and JW edited the manuscript. All authors revised the manuscript and read and approved the submitted version.

## Funding

This work was supported by grants from the Liaoning Provincial Natural Science Foundation of China (No.20170540251).

## Conflict of Interest

The authors declare that the research was conducted in the absence of any commercial or financial relationships that could be construed as a potential conflict of interest.

## Publisher’s Note

All claims expressed in this article are solely those of the authors and do not necessarily represent those of their affiliated organizations, or those of the publisher, the editors and the reviewers. Any product that may be evaluated in this article, or claim that may be made by its manufacturer, is not guaranteed or endorsed by the publisher.

## Abbreviations

TME, tumor immune microenvironment; NCAPG, non-structural maintenance chromatin condensin 1 complex subunit G; TMA, tissue microarray; CNS, central nervous system; BBB, blood brain barrier; MHC-I, major histocompatibility complex-I; DEG, differentially expressed gene; PPI, protein–protein interaction; STRING, search tool for the retrieval of interacting genes; IHC, immunohistochemistry; ROC, receiver operating characteristic; AUC, area under the curve; GSEA, gene set enrichment analysis; TIIC, tumor-infiltrating immune cell; IDH, isocitrate dehydrogenase; ADAM17, α-disintegrin and metalloprotease-17.
