# Supplementary material for: NCAPG Promotes Tumor Progression and Modulates Immune Cell Infiltration in Glioma
Source: Front Oncol. 2022 Mar 15;12:770628. doi: 10.3389/fonc.2022.770628 (PMC8964493; doi:10.3389/fonc.2022.770628)
Supplement: Supplementary file 1 [file DataSheet_1.docx]

Supplementary Material

# Supplementary Figure


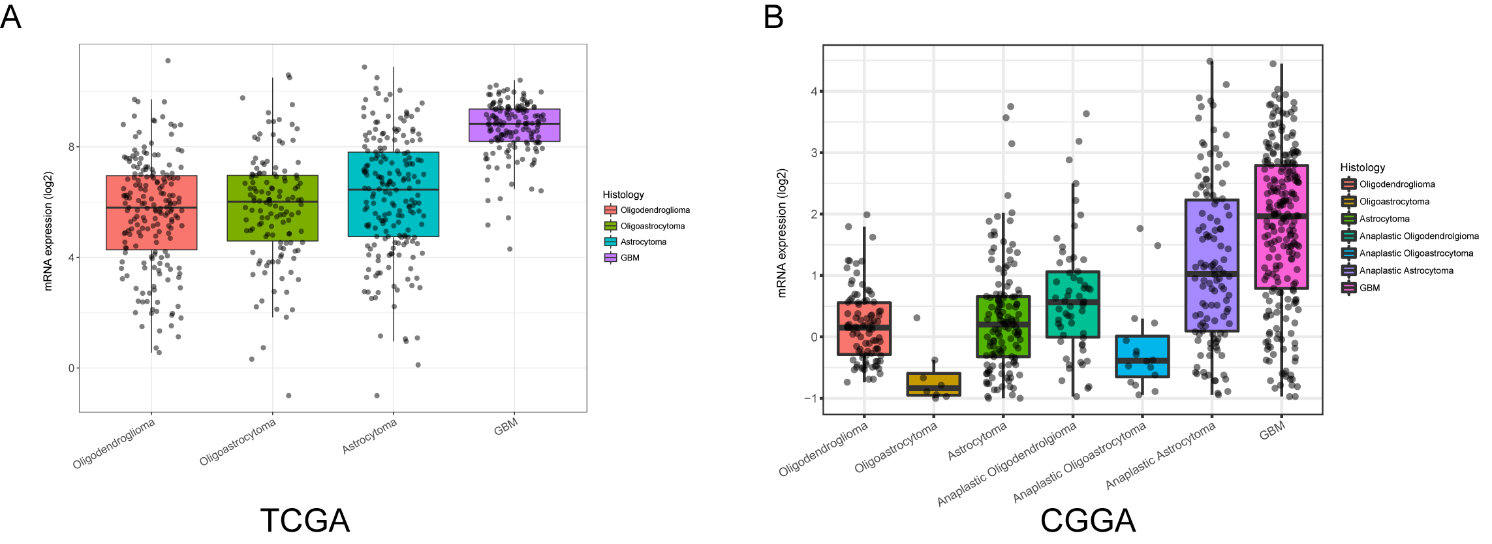


**Figure S1** The relationship between glioma histology subtypes and NCAPG mRNA expression levels. **(A)** Datasets from TCGA. **(B)** Datasets from CGGA.

# Supplementary Tables

**Table S1** siRNA sequences.

| **Name** | **Sense (5’-3’)** | **Antisense (5’-3’)** |
| --- | --- | --- |
| SiRNA-1 | GGAGUUCAUUCAUUACCUUTT | AAGGUAAUGAAUGAACUCCTT |
| SiRNA-2 | GCAAGGAAACACUUCGUAUTT | AUACGAAGUGUUUCCUUGCTT |
| SiRNA-3 | GGACUAAUCAGGAAUGCUUTT | AAGCAUUCCUGAUUAGUCCTT |

**Table S2** Primers for RT-qPCR.

| **Gene** | **Forward primers** | **Reverse primers** |
| --- | --- | --- |
| NCAPG | GAGGCTGCTGTCGATTAAGGA | AACTGTCTTATCATCCATCGTGC |
| HLA-A | AGACTGACCGAGTGGACCT | GTGATCTGAGCCGCCATGT |
| HLA-B | CAGTTCGTGAGGTTCGACAG | CAGCCGTACATGCTCTGGA |
| HLA-C | GGACCGGGAGACACAGAAGTA | CCTCGTTCAGGGCGATGTAAT |
| HLA-E | TTCCGAGTGAATCTGCGGAC | GTCGTAGGCGAACTGTTCATAC |
| HLA-G | GGTCGCAGCCAATCATCC | GAGGAGACACGGAACACCAAG |
| ADAM17 | GTGGATGGTAAAAACGAAAGCG | GGCTAGAACCCTAGAGTCAGG |
